# Intranasal Oxytocin Improves Lean Muscle Mass in Older Adults With Sarcopenic Obesity: A Pilot Study

**DOI:** 10.1093/geroni/igaa057.436

**Published:** 2020-12-16

**Authors:** Jessica Lee, Sara Espinoza, Adetutu Odejimi, Chen-pin Wang, Vinutha Ganapathy, Chiara Pascucci, Nicolas Musi, Elena Volpi

**Affiliations:** 1 The University of Texas Health Science Center at Houston, Houston, Texas, United States; 2 University of Texas Health Science Center at San Antonio, San Antonio, Texas, United States; 3 The University of Texas Medical Branch at Galveston, Galveston, Texas, United States; 4 Sapienza University of Rome, Rome, Italy

## Abstract

Obese older adults often have sarcopenia with increased functional impairments. Unfortunately, conventional weight loss treatments can lead to further muscle mass loss. Increasing evidence from animal studies suggests that the pituitary hormone oxytocin has trophic effects on skeletal muscle cells and can induce weight loss. We piloted a clinical trial testing whether intranasal oxytocin would decrease adiposity without lowering muscle mass in older adults with sarcopenic obesity. Twenty-one older (≥60years), obese (30-43kg/m2), sedentary (<2 strenuous exercises/week) adults with slow gait speed (<1m/sec) were randomized to intra-nasal oxytocin (24IU four times/day) or placebo for 8 weeks. Pre and post body mass index (BMI), 2-hour oral glucose tolerance test (OGTT), hemoglobin A1c (HbA1c), short physical performance battery (SPPB), and whole body lean and fat mass (via dual-energy X-ray absorptiometry) were assessed. Generalized estimation equation method was used to evaluate effects of oxytocin on these continuous measures. At baseline, results were: age 67.5±5.4years, 71% female, BMI 36.0±3.6kg/m2, HbA1c 5.7±0.4%, 2-hr OGTT glucose 140.8±4.1mg/dL, SPPB 9.2±1.9, fat mass 45,429±7,037g, and lean mass was 49,892±10,470g. From baseline to follow-up, total lean mass increased significantly (2,250g) in the oxytocin group (pre- vs. post-treatment difference of -690g in placebo and +1,559g in oxytocin, p<0.01). Oxytocin did not lead to significant changes in other measures. This data suggests that oxytocin leads to significant improvement in whole body lean mass. Future studies in a larger study population will help determine whether older adults with sarcopenic obesity may benefit from intranasal oxytocin to improve lean muscle mass and physical function.

